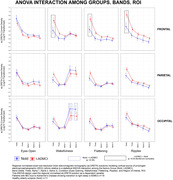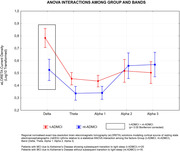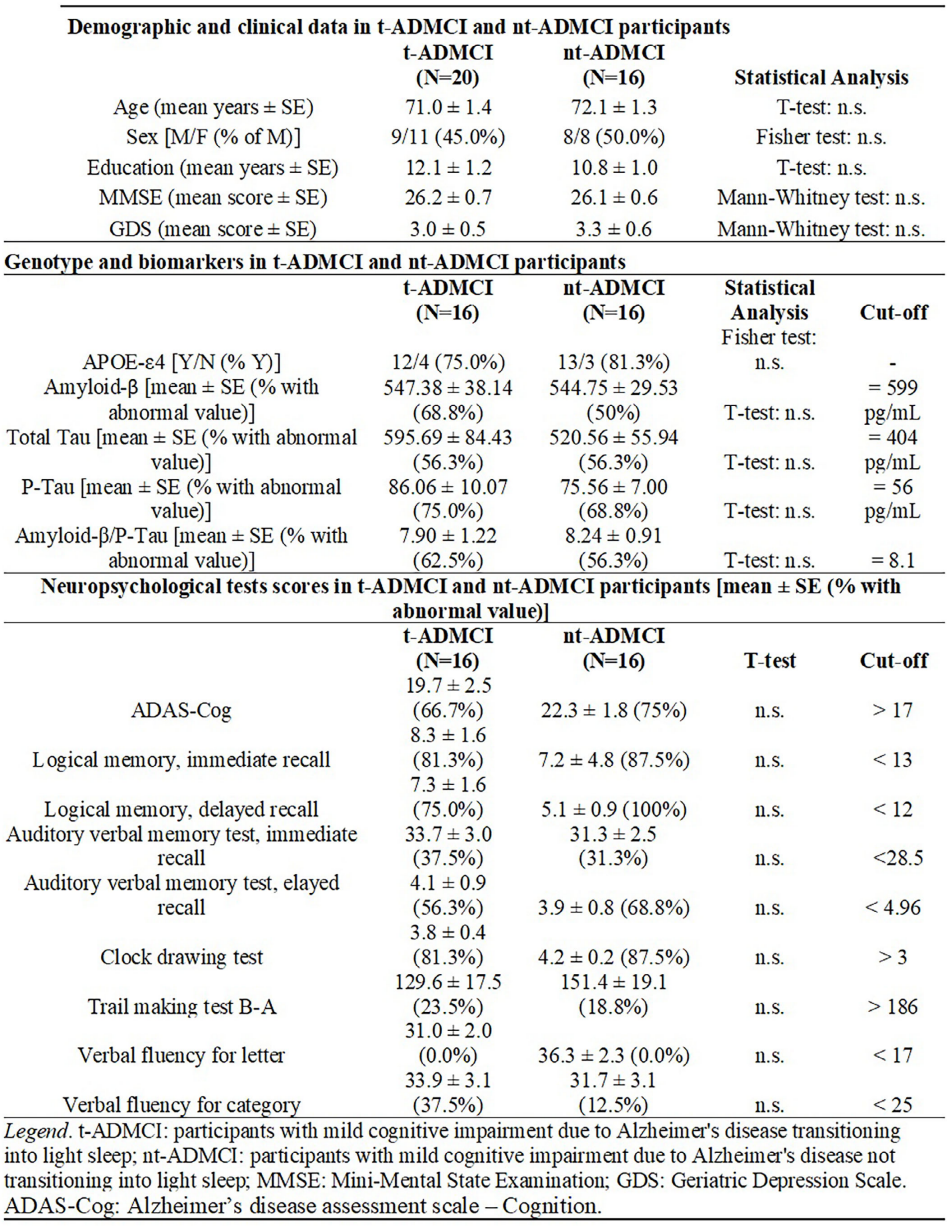# EEG Cortical Sources Abnormalities During Transition Between Wakefulness and Light Sleep in Patients with Alzheimer’s Disease and Mild Cognitive Impairment

**DOI:** 10.1002/alz.090667

**Published:** 2025-01-09

**Authors:** Enrico Michele Salamone, Matteo Carpi, Dharmendra Jakhar, Susanna Lopez, Giuseppe Noce, Roberta Lizio, Claudio Del Percio, Claudio Babiloni

**Affiliations:** ^1^ Sapienza University of Rome, Rome Italy; ^2^ IRCCS Synlab SDN, Naples Italy; ^3^ International Federation of Clinical Neurophysiology, Reston, VA USA; ^4^ San Raffaele Cassino, Cassino Italy

## Abstract

**Background:**

Alzheimer’s Disease (AD) and Sleep disturbances are closely linked in a bidirectional relationship. Indeed, sleep alterations represent a precipitating factor in early pathogenesis of neurodegeneration. Conversely, the accumulation of Aβ and tau proteins directly disrupts sleep‐wake cycles. Sleep disturbances are extremely prevalent in AD and the relationship between these disorders has been thoroughly investigated. Our study is the first to investigate the electroencephalographic cortical sources during the transitional stages between quiet wakefulness and light sleep in AD patients with MCI.

**Methods:**

We analysed data from an international database (www.pdwaves.org) comprising clinical, neuropsychological, EEG morning recordings (45 minutes), cerebrospinal fluid (CSF), structural and functional MRI data in n=36 ADMCI, n=20 showing transition to light sleep (t‐ADMCI) and n=16 without transition to sleep (nt‐ADMCI)(Table 1). The database also included clinical, neuropsychological, and EEG data of n=11 matched healthy elderly (Nold) persons transitioning to light sleep. The EEG traces were reviewed by researchers blinded to the diagnosis, divided in 4” epochs and categorised in four different vigilance conditions: Eyes Open, Wakefulness, Flattening of alpha (drowinsess), Ripples (light sleep), according to the sleep classification by Hori et al (1994). Individual alpha frequency (IAF) was used to determine the EEG delta, theta, and alpha bands. Regional EEG cortical sources were estimated using the eLORETA freeware.

**Results:**

Compared to Nold subjects, the ADMCI subjects showed greater Frontal Delta Cortical source activity in all vigilance conditions (p<0,001) (Figure 1). Furthermore, when compared to nt‐ADMCI, t‐ADMCI showed an increased diffuse delta cortical source activity (p<0.0001) during the wakefulness period (Figure 2). No differences between these two last groups emerged in CSF neurodegeneration biomarkers, neuropsychological testing nor structural or functional MRI measures.

**Conclusion:**

The higher Frontal Delta Cortical Source activity across all vigilance conditions in t‐ADMCI subjects as compared to Nold, and the persistence of this result when compared to matched nt‐ADMCI patients, likely reflects the presence of dominant local delta rhythms, normally found during N3 and REM sleep, due to a low dopaminergic and cholinergic tone. Our findings might reflect a “disconnection” of frontal regions due to the loss of projections from monoaminergic subcortical nuclei, affected by early neuronal loss from AD neuropathology.